# Validation of the City Birth Trauma Scale in a sample of Norwegian mothers

**DOI:** 10.1111/aogs.70149

**Published:** 2026-01-26

**Authors:** Silje Marie Haga, Luisa Bergunde, Lara Seefeld, Susan Ayers, Malin Eberhard‐Gran, Susan Garthus‐Niegel

**Affiliations:** ^1^ Regional Centre for Child and Adolescent Mental Health, Eastern and Southern Oslo Norway; ^2^ Institute and Policlinic of Occupational and Social Medicine, Faculty of Medicine and University, Hospital Carl Gustav Carus TUD Dresden University of Technology Dresden Germany; ^3^ Department of Psychotherapy and Psychosomatic Medicine, Faculty of Medicine and University, Hospital Carl Gustav Carus TUD Dresden University of Technology Dresden Germany; ^4^ Department of Psychology & Neuroscience, Life Sciences Centre Dalhousie University Halifax Canada; ^5^ School of Health & Medical Sciences City St. George's, University of London London UK; ^6^ Norwegian Research Centre for Women's Health Oslo University Hospital and University of Oslo Oslo Norway; ^7^ Institute for Clinical Medicine, Campus Ahus University of Oslo Lørenskog Norway; ^8^ Institute for Systems Medicine (ISM), Faculty of Medicine Medical School Hamburg, MSH Hamburg Germany; ^9^ Department of Childhood and Families Norwegian Institute of Public Health Oslo Norway

**Keywords:** birth trauma, childbirth‐related posttraumatic stress disorder, City Birth Trauma Scale

## Abstract

**Introduction:**

Approximately 3%–4% of women experience childbirth‐related posttraumatic stress disorder (CB‐PTSD). The City Birth Trauma Scale (City BiTS) is a questionnaire developed to assess CB‐PTSD, following the criteria outlined in the Diagnostic and Statistical Manual of Mental Disorders. The aim of the present study was to evaluate the psychometric properties of the Norwegian version of this questionnaire (City BiTS‐Nor).

**Material and Methods:**

A community sample of 1079 mothers completed a cross‐sectional online survey. The survey included questions on sociodemographic and obstetric characteristics, the City BiTS‐Nor, the Impact of Event Scale‐Revised, the Edinburgh Postnatal Depression Scale, the 10‐item anxiety subscale of the Hopkins Symptom Checklist, and the Bergen Insomnia Scale.

**Results:**

Confirmatory factor analysis supported a bifactor model comprising Birth‐related Symptoms and General Symptoms in CB‐PTSD, along with a General CB‐PTSD factor that explained 58.4% of the variance. The study found high internal consistency (≥0.90), and good convergent and divergent validity were shown. Discriminant validity was evaluated by examining factors such as mode of birth, maternal complications, infant complications, parity, history of traumatic childbirth, and previous traumatic experiences. Higher General and Birth‐related scores were observed in women who experienced emergency cesarean sections or instrumental vaginal births. This trend was observed in primiparous women, cases involving pregnancy and birth complications, and individuals with prior traumatic experiences.

**Conclusions:**

The City BiTS‐Nor presents appropriate psychometric properties for assessing CB‐PTSD symptoms according to DSM‐5 criteria. The findings suggest that using the total score, along with the individual subscale scores, is justified and enhances the comprehensive assessment of CB‐PTSD symptoms. These findings support the clinical utility of the City BiTS‐Nor as a screening tool for CB‐PTSD, with potential to differentiate childbirth‐related trauma from general psychopathology and to guide targeted interventions in perinatal care.

## Graphical abstract

The Norwegian City Birth Trauma Scale (City BiTS‐Nor) demonstrated strong psychometric properties in a sample of 1079 mothers. A bifactor structure and high reliability support its use for assessing childbirth‐related PTSD symptoms. Findings highlight its clinical utility for screening and guiding targeted interventions in perinatal mental health care.

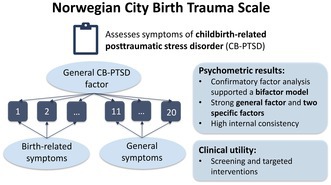

**Abbreviations**
City BiTS: City Birth Trauma Scale
CB‐PTSD: childbirth‐related posttraumatic stress disorder
DSM‐5: The Diagnostic and Statistical Manual of Mental Disorders, Fifth Edition
EPDS: Edinburgh Postnatal Depression Scale
PTSD: posttraumatic stress disorder

**Key message**

The Norwegian City Birth Trauma Scale (City BiTS‐Nor) demonstrates robust psychometric properties for assessing childbirth‐related PTSD, revealing an 8.7% prevalence. Our bifactor model distinguishes Birth‐related symptoms from General symptoms, providing clinicians with a validated tool for identifying affected mothers.

## 1 INTRODUCTION

Childbirth is a physically and emotionally intense experience with cultural significance.^1^ Women endure pain and physiological changes during labor^2^ while also navigating strong psychological reactions. When childbirth is a positive experience, it can empower women, strengthen their self‐esteem, and facilitate their adjustment to the maternal role.^3^

However, studies suggest that the prevalence rate of women who describe their birth as traumatic ranges from 9% to 45%^4^, ^5^, ^6^, ^7^, ^8^, ^9^ and around 3%–4% of women in community samples develop posttraumatic stress disorder after childbirth (CB‐PTSD).^1^, ^10^, ^11^, ^12^ A birth is considered traumatic if a woman experiences intense feelings of helplessness, fear, or loss of control.^13^, ^14^ The subjective birth experience is a primary factor contributing to the traumatic effect and development of posttraumatic stress symptoms.^15^ This means that births considered normal from a medical perspective can still result in psychological trauma, although the risk of birth trauma tends to increase with the frequency of medical interventions.^16^, ^17^ Symptoms of CB‐PTSD include intrusions related to the birth experience, avoidance of trauma‐related stimuli, mood and cognitive alterations, and symptoms of hyperarousal.^18^

A traumatic childbirth can lead to mental health challenges,^13^, ^19^ identity issues,^20^ breastfeeding problems,^21^ difficulties in the mother–child and partner relationships,^22^, ^23^, ^24^, ^25^, ^26^ and reduced planning for future children.^27^ Intergenerational transmission of stress and trauma further emphasizes the extensive consequences not only for the mother but also for her children.^28^, ^29^ Consequently, CB‐PTSD is a significant public health issue with considerable economic implications for society.^15^, ^30^, ^31^ In Norway, routine postpartum care is lacking a systematic assessment of CB‐PTSD.

The City Birth Trauma Scale (City BiTS) is a 29‐item self‐report questionnaire designed to measure CB‐PTSD based on The Diagnostic and Statistical Manual of Mental Disorders (DSM‐5) criteria.^32^ It has a two‐factor structure: Birth‐related symptoms (symptoms of intrusion, avoidance, and negative cognitions and mood) and General symptoms (symptoms of hyperarousal and other symptoms of negative cognitions and mood), which have been replicated in international studies.^33^, ^34^, ^35^, ^36^, ^37^, ^38^, ^39^, ^40^, ^41^, ^42^, ^43^ Recent studies explore a bifactor model, highlighting the importance of subscale scores alongside the total score.^35^, ^37^, ^40^, ^41^ Given that PTSD has four clusters according to the DSM‐5 and a systematic review of PTSD's latent structure indicated that the disorder's symptom structure is better captured by multiple lower order models (e.g., six‐ and seven‐factor models),^44^ this study aimed to compare the lower order two‐factor and four‐factor models of the City BiTS with the bifactor model. To date, the psychometric properties of the Norwegian City BiTS have not been examined.

### 1.1 Current study

This study aimed to assess the psychometric properties of the Norwegian version of the City BiTS for measuring CB‐PTSD in Norwegian mothers. It was hypothesized that City BiTS‐Nor would demonstrate satisfactory validity and reliability. We expected a two‐factor model (Birth‐related and General symptoms) to outperform a four‐factor model, as seen in prior studies.^32^, ^34^, ^36^, ^43^ Additionally, we anticipated support for a bifactor model, including a General CB‐PTSD factor alongside the two subscales, consistent with findings from Croatia, France, and Australia.^35^, ^37^, ^41^

## 2 MATERIAL AND METHODS

### 2.1 Study population and design

Postpartum women in Norway participated in an online cross‐sectional study to validate the City BiTS‐Nor. Recruitment occurred in Maternal and Child Health‐Care‐Centers during routine consultation, through social media, and in Oslo through in‐person outreach. Eligible participants were women aged 18+ who had given birth in the past year with the birth being at least 1 month ago. Data collection ran from August 2022 to December 2023. The current study utilizes data collected up until August 2023. By then, a total of 1079 women who met the inclusion criteria had provided informed consent to participate. Ethical approval was granted by the Norwegian Regional Committees for Medical and Health Research Ethics (project 275370), and the study was preregistered at https://osf.io/stbqj/.

### 2.2 Instruments

Sociodemographic and obstetric characteristics included age, ethnicity, relationship status, education, length of gestation, infant age, parity, mode of birth, maternal and infant complications during pregnancy or birth, whether the pregnancy was planned, preterm birth, and any history of birth trauma (see Table 1 for details).

**TABLE 1:** Sample descriptive characteristics (*N* = 1079).

	*Mean* (SD), range or *n* (%)
*Sociodemographic variables*	
Maternal age in years *Mean* (SD), range	31.72 (4.24), 19–47
Ethnicity Majority Norwegian *n* (%)	1035 (95.9)
Relationship status *n* (%)	
In a couple relationship	1057 (98.0)
Single, separated, divorced, or widowed	22 (2.0)
Education level *n* (%)	
Compulsory education or primary school or less	13 (1.2)
Postcompulsory education (e.g., secondary school)	134 (12.4)
Higher education (e.g., university)	932 (86.4)
First‐time mother *n* (%)	632 (58.6)
History of birth trauma^a^ *n* (%)	164 (15.2)
Length of gestation in weeks *Mean* (SD), range	39.58 (1.93), 26–42
Preterm birth^b^ *n* (%)	57 (5.3)
Planned pregnancy *n* (%)	947 (87.8)
Mode of birth *n* (%)	
Spontaneous vaginal birth	741 (68.7)
Instrumental vaginal birth	149 (13.8)
Emergency cesarean section	146 (13.5)
Planned cesarean section	43 (4.0)
Maternal complications during pregnancy/birth *n* (%)	450 (41.7)
Infant complications during pregnancy/birth *n* (%)	209 (19.4)
Infant age (days) *Mean* (SD), range	156.05 (84.56), 29–364

^a^ Answered by *n* = 447 multiparous women.

^b^ Preterm birth defined as <37 weeks gestation.

Previous trauma was assessed using a 14‐item checklist, with eight questions adapted from the Posttraumatic Stress Diagnostic Scale,^45^ a reliable and valid tool^46^ used in perinatal research.^47^, ^48^ The checklist covers physical, sexual, and psychological assault, domestic violence, serious accidents, and an “other” category. Five additional items assess emotional neglect, substance abuse, mental health issues, suicidal behavior, and family imprisonment. Participants endorsing any item are coded as “1” (history of trauma), while others are coded “0” (no trauma history).

CB‐PTSD was assessed using the City BiTS, a 29‐item questionnaire based on DSM‐5 criteria.^32^, ^49^ The stressor criterion was first evaluated through two yes/no questions on perceived threats to the woman or baby. Symptom frequency in the past week was then measured across four categories—intrusion (five items), avoidance (two items), negative cognitions/mood (seven items), and hyperarousal (six items)—using a four‐point scale (0–3), with a total score ranging from 0 to 60. Two items screened for a dissociative subtype, while five additional items assessed symptom onset, duration, distress, impairment, and exclusion criteria (e.g., effects of medication or illness). The original study identified two subscales: Birth‐related symptoms (10 items) and General symptoms (10 items). With permission from Susan Ayers, the City BiTS was translated into Norwegian using the back‐translation method.^50^

CB‐PTSD symptoms were also measured using the Norwegian version of the Impact of Event Scale‐Revised (IES‐R),^51^ a 22‐item self‐rating scale that measures symptoms of intrusion (seven items), avoidance (eight items), and hyperarousal (seven items), based on the DSM‐IV.^52^ Participants rated the items in relation to their childbirth experience. Responses are given on a five‐point scale (0 = not at all, 1 = rarely, 2 = sometimes, 3 = often, and 4 = very often) and reflect distress experienced in the past 7 days.

Postpartum depressive symptoms were assessed using the Norwegian Edinburgh Postnatal Depression Scale (EPDS),^53^ the most widely used screening tool for perinatal depression.^54^, ^55^ The 10‐item scale rates symptoms from the past week on a four‐point scale (0–3), with total scores ranging from 0 to 30. Scores ≥10 indicate minor depression, while ≥12 suggest moderate depression.^55^

Anxiety symptoms were assessed using the Norwegian translation of the 10‐item anxiety subscale of the Hopkins Symptom Checklist (HSCL‐25).^56^ Each item is rated on a scale from 1 (not bothered) to 4 (extremely bothered), with higher scores reflecting greater levels of anxiety. The total score is calculated as the mean score, with a range from 1 to 4.

Insomnia was assessed using the Bergen Insomnia Scale (BIS),^57^ 2008, a six‐item self‐report tool based on DSM‐IV‐TR criteria.^58^ It evaluates sleep onset, maintenance, early waking, restfulness, daytime impairment, and sleep dissatisfaction over the past month. Responses are rated on an eight‐point scale (0–7 days per week), with total scores ranging from 0 to 42.

### 2.3 Statistical analysis

All analyses were conducted using IBM® SPSS® version 29, except for the confirmatory factor analysis (CFA), which was performed using Mplus 8.1 software,^59^ and calculations of omega hierarchical and omega total, which was done in R version 4.3.2.^60^ Significance was set at *p* < 0.05. Preanalysis steps for conducting CFA involved checking skew (≤2) and kurtosis (≤7) of the City BiTS‐Nor items and screening the data for multivariate outliers via Mahalanobis distance, with an alpha level of 0.001.^61^ We excluded 77 participants identified as multivariate outliers for CFA analysis (CFA sample *N* = 1002) but used the full sample (*N* = 1079) for all other analyses as symptoms are often skewed in non‐clinical samples. Since three items (b2, c2, e2) exceeded skewness and kurtosis cutoffs and the City BiTS items are ordinal, we used the diagonally weighted least squares (WLSMV) estimator, which is specifically designed to handle non‐normal and ordinal data.^62^, ^63^ Model fit was evaluated using the following fit indices and their respective cutoffs for good fit^64^: Root Mean Square Error of Approximation (RMSEA <0.06), Comparative Fit Index (CFI >0.95), Tucker‐Lewis Index (TLI >0.95), and Standardized Root Mean Square Residual (SRMR <0.08). A nonsignificant $\chi^{2}$ value reflects good fit, however in large samples like this study, nonsignificant *p*‐values are less likely,^65^ therefore we used the statistic adjusted for its degrees of freedom to indicate fit ($\chi^{2}/\mathrm{df}$ < 5).^66^ Additionally, to evaluate the bifactor model, we reported the explained common variance (ECV), which describes the common variance explained by the general factor divided by the total common variance.^67^

Reliability of the City BiTS‐Nor total and subscale scores was assessed using Cronbach's alpha coefficient (α), with α of 0.70 or greater considered indicative of acceptable internal reliability.^68^ Since α may underestimate reliability when items are not tau‐equivalent,^69^ we also report McDonald's omega reliability statistic,^70^ calculated using the Hayes Omega Macro in SPSS.^71^ Additionally, we provide omega hierarchical and total statistics for the total score to estimate the general factor saturation. Calculation of omega hierarchical and total test statistics is based on an exploratory factor analysis model derived from a minimum residual factor analysis of the dataset, to which the Schmid–Leiman transformation is applied, generating general factor loadings.^72^ Omega hierarchical gives a reliability estimate for the variance in the data that is attributed solely to the general factor, while omega total considers both the general and specific factors.

Construct validity was tested by correlating the DSM‐5 City BiTS‐Nor subscales of “intrusions”, “avoidance”, and “hyperarousal” with the corresponding IES‐R subscales, the City BiTS‐Nor subscale “negative cognitions and mood” with the sum‐score of EPDS, and the “hyperarousal” items with the BIS. Convergent validity (IES‐R) and divergent validity (EPDS, HSCL‐25) were analyzed using Pearson's correlation coefficient. Correlation coefficients were interpreted as follows: *r* < 0.30 (negligible), *r* ≥ 0.30 (low), *r* ≥ 0.50 (moderate), *r* ≥ 0.70 (high), *r* ≥ 0.90 (very high).^73^ We further examined discriminant validity regarding mode of birth, maternal and infant complications during pregnancy or birth, parity, history of traumatic childbirth, and previous traumatic experiences. This was done for the total and subscale scores of the City BiTS‐Nor using one‐way ANOVAs and independent samples Welch *t*‐tests, the latter being recommended for its robustness compared to the standard *t*‐test. If the assumption of homogeneity of variance was violated (Levene's test *p* < 0.05), we interpreted Welch ANOVA and Games‐Howell posthoc test results; we otherwise relied on Sidak‐corrected posthoc tests.

## 3 RESULTS

### 3.1 Sample characteristics

The study included 1079 mothers (see Table 1). Their average age was 31.72 years, with most in a couple relationship. The majority (86.4%) had higher education, and nearly 58.6% were first‐time mothers. Most births were spontaneous vaginal, while 17.5% were cesarean and 5.3% were preterm.

Table 2 summarizes City BiTS‐Nor responses, indicating that 21.8% of participants met the stressor criterion (i.e., they believed that they or their baby would be seriously injured or die during or immediately after childbirth). Overall, 94 mothers (8.7%) met all DSM‐5 criteria for a PTSD diagnosis, with 83 (7.7%) classified as incidental cases.

**TABLE 2:** Assessment of CB‐PTSD using City BiTS‐Nor DSM‐5 diagnostic criteria (*N* = 1079).

Criterion		*n*, %
A	Stressor criterion (“yes” on item 1 or item 2)	235 (21.8)
B	Intrusion symptoms (1 needed)	642 (59.5)
C	Avoidance symptoms (1 needed)	264 (24.5)
D	Negative cognitions and mood (2 needed)	667 (61.8)
E	Hyperarousal (2 needed)	776 (71.9)
F	Duration (>1 month necessary)	635 (58.9)
G	Distress and impairment (“sometimes” or “yes” on item 27 or item 28)	659 (61.1)
H	Exclusion criteria (“maybe” or “yes” on item 29)	34 (4.4)
	Dissociative symptoms (1 needed for dissociative subtype)	400 (37.1)
	Diagnostic criteria met for CB‐PTSD	94 (8.7)
	…thereof with dissociative symptoms	73 (6.7)
	…thereof with incidental symptoms after childbirth	83 (7.7)

*Note*: Item 1 = “Did you believe you or your baby would be seriously injured?” Item 2 = “Did you believe you or your baby would die?” Item 27 = “Do these symptoms cause you a lot of distress?” Item 28 = “Do they prevent you doing things you usually do (e.g. socializing, daily activities)?” Item 29 = “Could any of these symptoms be due to medication, alcohol, drugs, or physical illness?”

Abbreviations: BiTS‐Nor, Norwegian City Birth Trauma Scale; CB‐PTSD, childbirth‐related posttraumatic stress disorder; DSM‐5, diagnostic and statistical manual of mental disorders.

### 3.2 Confirmatory factor analysis

The four‐factor model, which included the factors intrusions, avoidance, negative cognitions and mood, and hyperarousal, yielded a poor model fit, $\chi^{2}$_(164)_ = 2656.452, $\chi^{2}/\mathrm{df}$ = 16.20, RMSEA = 0.123, SRMR = 0.125, CFI = 0.912, TLI = 0.898. Additionally, higher correlations among factors also indicated a linear dependency. The intrusions factor explained 28% of the items' variance, the avoidance factor 13%, the negative cognitions/mood factor 31%, and the hyperarousal factor 28%.

The two‐factor model, including the correlated Birth‐related symptoms and General symptoms factors, showed better model fit regarding all fit indices, $\chi^{2}$_(169)_ = 851.321, $\chi^{2}/\mathrm{df}$ = 5.04, RMSEA = 0.063, SRMR = 0.056, CFI = 0.976, TLI = 0.973 (see Table 3 for standardized factor loadings for the two‐factor model). Although the RMSEA slightly exceeded the 0.06 cutoff, all other fit indices indicated good fit. A moderate correlation (*r* = 0.51) was observed between Birth‐related symptoms and General symptoms. The Birth‐related symptoms factor and the General symptoms factor accounted for 48% and 52% of the total item variance, respectively.

**TABLE 3:** Standardized Factor Loadings for the Two‐Factor and Bifactor Model of the City BiTS‐Nor (*n* = 1072).

Items	Two‐factor model		Bifactor model		
	BRS	GS	G	BRS	GS
**Birth‐related symptoms**					
*Intrusions (1–5)*					
1. Recurrent unwanted memories of the birth	0.85		0.667	0.617	
2. Bad dreams or nightmare about the birth	0.90		0.848	0.311	
3. Flashbacks to the birth and/or reliving the experience	0.73		0.480	0.759	
4. Getting upset when reminded of the birth	0.94		0.896	0.294	
5. Feeling tense or anxious when reminded of the birth	0.96		0.928	0.264	
*Avoidance (6–7)*					
6. Trying to avoid thinking about the birth.	0.92		0.901	0.189	
7. Trying to avoid things that remind me of the birth.	0.88		0.896	0.069	
*Negative cognitions and mood (8–14)*					
8. Not able to remember details of the birth.	0.47		0.465	0.078	
9. Blaming myself or others for what happened during the birth.	0.79		0.769	0.178	
10. Feeling strong negative emotions about the birth.	0.93		0.888	0.284	
**General symptoms**					
11. Feeling negative about myself or … will happen.		0.81	0.505		0.623
12. Lost interest in activities that were important to me.		0.86	0.472		0.717
13. Feeling detached from other people.		0.87	0.463		0.743
14. Not able to feel positive emotions.		0.86	0.423		0.756
*Hyperarousal (15–20)*					
15. Feeling irritable or aggressive.		0.83	0.341		0.784
16. Feeling self‐destructive or acting recklessly.		0.75	0.468		0.570
17. Feeling tense or on edge.		0.83	0.350		0.781
18. Feeling jumpy or easily startled.		0.72	0.466		0.537
19. Problems concentrating.		0.79	0.405		0.682
20. Not sleeping well… not due to the baby's sleep pattern.		0.76	0.568		0.475

Abbreviations: BiTS‐Nor, Norwegian City Birth Trauma Scale; BRS, Birth‐related symptoms; G, General CB‐PTSD factor; GS, General symptoms.

The bifactor model, which included a General CB‐PTSD factor along with the Birth‐related symptoms and General symptoms factors showed the best fit across all indices: $\chi^{2}$_(150)_ = 479.728, $\chi^{2}/\mathrm{df}$ = 3.20, RMSEA = 0.047, SRMR = 0.031, CFI = 0.988, TLI = 0.985 (see Table 3 for standardized factor loadings). The ECV indices indicated that a significant portion of the shared variance among the 20 items (58.4%) was attributed to the General CB‐PTSD factor, supporting the presence of a strong general factor. Within this, Birth‐related symptoms contributed 9.6%, while the General symptoms factor accounted for 32%.

CFA results were also consistent when including multivariate outliers (data not shown).

### 3.3 Reliability

Cronbach's alphas of the total City BiTS‐Nor score, as well as the Birth‐related symptoms and General symptoms scores, were all excellent (≥0.90; Blanz,^68^ see Table 4). Examining item deletion effects revealed no item redundancy for the total and subscale scores. McDonald's omega was also excellent for both total and subscale scores. The hierarchical omega statistic was 0.57, and the omega total statistic was 0.94 (Table 4).

**TABLE 4:** Results from reliability analyses using Cronbach's alpha and omega.

	Cronbach's alpha^a^ α	McDonald's omega^a^ *ω*	Hierarchical omega^b^ *ω*_*h*_	Total omega^b^ *ω*_*t*_
Total score	0.91	0.90	0.57	0.94
Birth‐related symptoms score	0.91	0.91		
General symptoms score	0.90	0.90		

^a^ Calculated with Hayes Macro in SPSS.^71^

^b^ Calculated in R version 4.3.2 using omega in the *psych* package.^74^

### 3.4 Construct, convergent, and divergent validity

Construct validity was assessed by correlating the DSM‐5 subscales (intrusions, avoidance, hyperarousal) of the City BiTS against the respective IES‐R subscales. Intrusion subscales (*r* = 0.78, *p* < 0.001), avoidance subscales (*r* = 0.72, *p* < 0.001), and hyperarousal subscales (*r* = 0.60, *p* < 0.001) showed high and moderate correlations, respectively. Furthermore, the City BiTS subscale “negative cognitions and mood” was correlated against the EPDS (*r* = 0.67, *p* < 0.001), and the “hyperarousal” items against the BIS (*r* = 0.38, *p* < 0.001), evidencing moderate and low correlations, respectively.

Convergent validity, tested via correlations between the City BiTS‐Nor and the IES‐R, was supported by high correlations between the IES‐R and both the City BiTS‐Nor total scale and the Birth‐related symptoms subscale, as well as moderate correlations with the General symptoms subscale (Table 5).

**TABLE 5:** Convergent and divergent validity results with Pearson correlations between the City BiTS‐Nor total and subscale scores with IES‐R, EPDS, and HSCL‐25.

Variable	*M* (*SD*)	2	3	4	5	6	7
1. City BiTS‐Nor total score	14.72 (12.47)	0.81**	0.88**	0.79**	0.68**	0.66**	0.42**
2. CityBiTS‐Nor Birth‐related symptoms	5.33 (6.61)	–	0.44**	0.79**	0.44**	0.48**	0.32**
3. City BiTS‐Nor General symptoms	9.39 (8.08)		–	0.57**	0.69**	0.63**	0.39**
4. IES‐R	10.91 (13.58)			–	0.62**	0.65**	0.40**
5. EPDS	5.67 (4.85)				–	0.74**	0.45**
6. HSCL‐25	1.38 (0.42)					–	0.42**
7. BIS	15.98 (9.56)						–

Abbreviations: BIS, Bergen Insomnia Scale; City BiTS‐Nor, Norwegian version of the City Birth Trauma Scale; EPDS, Edinburgh Postnatal Depression Scale; HSCL‐25, Anxiety Subscale of Hopkins Symptom Checklist; IES‐R, Impact of Event Scale Revised; M, mean; SD, standard deviation.

** *p* < 0.001.

Divergent validity was assessed by correlating the City BiTS‐Nor with the EPDS and HSCL‐25. The City BiTS‐Nor showed moderate correlations with the EPDS and the HSCL‐A. The EPDS and HSCL‐A total scores exhibited low correlations with the Birth‐related subscale and moderate correlation with the General symptoms subscale (Table 5).

### 3.5 Discriminant validity

Discriminant validity of the City BiTS‐Nor total score and its subscale scores (Birth‐related symptoms and General symptoms) were assessed via analysis of known‐group differences (Table 6). A one‐way ANOVA revealed significant differences in total CB‐PTSD symptoms, Birth‐related symptoms, and General symptoms. Games‐Howell posthoc analysis showed that individuals with spontaneous vaginal birth reported significantly fewer overall CB‐PTSD symptoms compared to those who experienced instrumental vaginal birth and an emergency cesarean section. Similarly, Games–Howell posthoc tests showed that women with spontaneous vaginal birth had significantly lower Birth‐related symptoms scores than those with instrumental vaginal birth and emergency cesarean section. However, Sidak‐corrected posthoc tests revealed no significant differences in General symptoms scores based on mode of birth (*p* > 0.053). Welch *t*‐tests further demonstrated that women who reported maternal complications during pregnancy or birth, infant complications during pregnancy or birth, were primiparous, had previously experienced a traumatic childbirth, or had experienced at least one traumatic event had elevated total CB‐PTSD symptoms, Birth‐related symptoms, and General symptoms. The only exception was that General symptoms scores did not differ significantly with parity (Table 6).

**TABLE 6:** Discriminant validity results via known group differences with *t*‐test and ANOVA examining the City BiTS‐Nor total and subscale scores.

	*n*	Total score	Birth‐related symptoms	General symptoms
		*Mean* (SD)	*Mean* (SD)	*Mean* (SD)
Mode of birth				
Spontaneous vaginal birth	741	13.21 (11.22)	4.16 (5.60)	9.05 (7.81)
Instrumental vaginal birth	149	17.87 (14.31)	7.21 (8.06)	10.65 (8.34)
Emergency c‐section	146	19.71 (13.90)	9.25 (7.56)	10.45 (8.51)
Planned c‐section	43	17.79 (15.81)	5.88 (8.49)	11.91 (9.39)
		*F*(3, 147.5) = 13.33** ^,^ ^a^	*F*(3, 145.51) = 24.58** ^,^ ^a^	*F*(3,1075) = 3.71* ^,^ ^b^
Maternal complications during pregnancy/birth^c^				
No	597	12.37 (11.14)	3.91 (5.42)	8.46 (7.81)
Yes	450	18.09 (13.44)	7.11 (7.57)	10.98 (8.21)
		*t*(860.6) = −7.34**	*t*(776.3) = −7.62**	*t*(940.9) = −5.03**
Infant complications during pregnancy/birth^c^				
No	934	13.94 (12.07)	4.79 (6.28)	9.16 (7.94)
Yes	220	18.93 (13.57)	7.61 (7.69)	11.33 (8.43)
		*t*(292.1) = −4.87**	*t*(278.3) = −4.92**	*t*(302.8) = −3.38**
Parity^c^				
Primiparous	632	15.86 (12.67)	6.10 (6.87)	9.76 (8.10)
Multiparous	447	13.58 (12.20)	4.26 (6.22)	9.32 (8.05)
		*t*(982.1) = 2.97*	*t*(1014.1) = 4.57**	*t*(963.9) = 0.89
Previous traumatic birth^c^, ^d^				
No	283	11.48 (10.23)	2.82 (4.22)	8.67 (7.83)
Yes	164	17.20 (14.34)	6.76 (8.06)	10.43 (8.33)
		*t*(260.1) = −4.48**	*t*(215.7) = −5.82**	*t*(323.46) = −2.21*
Previous trauma^c^				
No	450	12.34 (11.31)	4.27 (5.77)	8.06 (7.50)
Yes	629	16.76 (13.02)	6.10 (7.14)	10.66 (8.31)
		*t*(1037.3) = −5.94**	*t*(1061.0) = −4.63**	*t*(1020.5) = −5.35**

^a^ Welch ANOVA results and Games‐Howell post‐hoc tests are interpreted due to the significant Levene's test and violation of the assumption of homogeneity of variance.

^b^ Univariate ANOVA and Sidak post‐hoc tests are interpreted due to non‐significant Levene's test.

^c^ Welch *t*‐test is reported as it is considered more robust than the *t*‐test.

^d^ *n* = 447 as only multiparous women answered this question.

* *p* < 0.05.

** *p* < 0.001.

## 4 DISCUSSION

The present study investigated the psychometric properties of the City BiTS‐Nor in a community sample of Norwegian mothers. The findings confirm the instrument's reliability and validity, offering valuable insights into its factor structure and practical applicability.

Regarding factor structure, our study provides strong evidence for a bifactor model consisting of a General CB‐PTSD factor along with specific factors for Birth‐related symptoms and General symptoms. This model demonstrated an even better fit than the two‐factor model originally proposed by Ayers, Wright^32^ and Weigl, Beck‐Hiestermann^43^ and is consistent with recent research.^35^, ^37^, ^41^ In this model, the General CB‐PTSD factor explained 58.4% of the shared variance, with General symptoms accounting for 32% and birth‐related symptoms for 9.6%. This distribution mirrors findings involving French‐speaking mothers^41^ and highlights the importance of the total score, while also supporting the utility of subscales as differentiated assessment. No support was found for the four‐factor model based on DSM‐5 criteria symptom clusters.

The psychometric properties of the City BiTS‐Nor proved robust. Internal consistency was excellent throughout the study, as evidenced by both Cronbach's alpha and McDonald's omega coefficient. Construct validity was confirmed through substantial correlations with IES‐R subscales, the EPDS, and—investigated for the first time—with insomnia (BIS). Convergent validity was demonstrated by high correlations between the IES‐R and both the City BiTS‐Nor total score and Birth‐related symptoms subscale. In contrast, moderate correlations were observed with the General symptoms subscale. This pattern supports the distinctiveness of the subscales and their unique contributions to the assessment. Divergent validity was further evidenced by the differential correlation patterns of the subscales: General symptoms showed stronger correlations with depression (EPDS) and anxiety symptoms (SCL‐A) than Birth‐related symptoms. This underscores the value of separately assessing both symptom domains and suggests that the Birth‐related symptoms subscale captures a distinct aspect of CB‐PTSD that is less related to general psychopathology, consistent with findings in Croatia^37^ and France.^41^ Thus, it is important to acknowledge that the total score of the City BiTS encompasses considerable overlap with general psychopathological symptoms. In light of the high comorbidity between postpartum depressive symptoms and CB‐PTSD,^75^, ^76^ this highlights the importance of comprehensive psychometric and subscale assessments to guide targeted therapeutic interventions and lends support to the appropriateness of a transdiagnostic approach to psychopathology in the postpartum period.^77^

Discriminant validity was confirmed through significant differences between known risk groups. As expected, both higher total scores were observed in women who underwent emergency cesarean section and instrumental vaginal birth compared to those with a spontaneous vaginal birth, in primiparous women, in cases with pregnancy/birth complications, and in individuals with previous traumatic experiences. These findings replicate and extend previous studies,^35^, ^36^, ^37^, ^41^, ^43^ underscoring the clinical validity of the instrument. Interestingly, the General symptoms subscale did not differentiate significantly regarding birth mode or parity, suggesting that the Birth‐related symptoms subscale may reflect a partially distinct symptom cluster that may be more sensitive and help identify individuals at risk. This highlights the utility of including birth‐specific symptom items in screenings, which may further inform clinical interventions.

The prevalence of CB‐PTSD in our sample was 8.7%, which is higher than the meta‐analytic findings of 4.7% reported by Heyne and colleagues,^12^ but comparable to other online validation studies of the City BiTS (12.5% in Australia^35^; 11.8% in Croatia^37^). Moreover, 22% experienced birth as traumatic according to DSM‐5 criteria. Though recruitment to the current study was conducted via healthcare networks, social media, community outreach, and research panels—methods that align with those used in other City BiTS validation studies—there remains a possibility that the elevated prevalence may be influenced by selection effects inherent to online recruitment strategies. Further cross‐national research is warranted to investigate cultural, systemic, and contextual factors that could account for observed differences in prevalence rates. Future studies should specifically aim to elucidate country‐level variations in both the development and the reporting of CB‐PTSD, to better understand how national contexts shape maternal mental health outcomes.

The present study has several notable strengths. First, it utilized a large sample of over 1000 mothers, providing robust statistical power for the psychometric analyses. Second, our comprehensive validation approach included multiple measures of reliability and validity, including the calculation of McDonald's omega alongside Cronbach's alpha, which is particularly valuable considering the non‐tau‐equivalence of scale items. Third, we were the first to explore the relationship between CB‐PTSD symptoms and insomnia using the BIS, thereby enhancing our understanding of the scale's construct validity. Fourth, our study compared different factor models, including the bifactor model, providing strong evidence for the optimal scoring structure of the City BiTS. Lastly, the inclusion of various risk groups allowed for a thorough examination of the scale's discriminant validity.

However, several limitations should be acknowledged. First, our sample predominantly comprised highly educated women, which may limit the generalizability of the findings to the broader population. Additionally, the cross‐sectional design of the study precludes conclusions about the temporal stability of CB‐PTSD symptoms and the scale's sensitivity to change over time. Given that time since the birth varied between 1 and 12 months in our sample, it should also be considered that this may have affected symptom scores in light of research suggesting temporal variation in CB‐PTSD symptoms.^78^

The findings of this study have important implications for both research and clinical practice. From a research perspective, this validation study is part of the International Survey of Childbirth‐Related Trauma (INTERSECT; https://www.researchregistry.com/browse‐the‐registry#home/registrationdetails/5ffc7453702012001b80a58c/). The survey aims to understand how many women experience birth trauma and PTSD in different countries and cultures, and how CB‐PTSD symptoms may vary in these different contexts and health care settings, using the City BiTS. Our findings contribute to this broader international effort to establish the cross‐cultural validity and applicability of the scale. In the Norwegian context, an important next step is to validate a partner version of the City BiTS‐Nor, a project currently underway by our team. This will help determine prevalence rates among fathers/partners in Norway and investigate why these prevalence rates are approximately four times lower among fathers than those among mothers.^79^ Furthermore, future research should focus on establishing clinical cutoff scores, assessing the scale's predictive validity, and conducting longitudinal studies to better understand the trajectories of CB‐PTSD symptoms over time. This is crucial given the potential long‐term health implications for women, their infants, and families.^25^, ^28^, ^76^, ^80^, ^81^, ^82^

For clinical practice, our results endorse the use of both total and subscale scores of the City BiTS‐Nor for a comprehensive assessment of CB‐PTSD symptoms. The scale's ability to differentiate between risk groups (e.g., emergency cesarean section, pregnancy/birth complications) makes it particularly valuable for targeted screening in routine postpartum care. This is especially important given that Norway currently lacks systematic assessment of CB‐PTSD.

Given the higher prevalence of CB‐PTSD found in our sample compared to meta‐analytic estimates, there is a clear need for systematic assessment within the Norwegian healthcare context. This necessitates the implementation of validated screening tools and appropriate training for healthcare professionals to recognize and respond to birth trauma effectively. Early identification and intervention for CB‐PTSD are crucial in preventing the development of chronic symptoms and mitigating their personal and familial impacts.^80^

## 5 CONCLUSION

The City BiTS‐Nor demonstrates robust psychometric properties, establishing itself as a reliable and valid instrument for assessing CB‐PTSD symptoms in Norwegian mothers. Our findings support the bifactor structure of the scale encompassing a General CB‐PTSD factor as well as Birth‐related and General symptom factors. This offers clinicians and researchers a nuanced approach to assessment. The scale's capacity to distinguish between known risk groups, combined with its robust psychometric properties, makes it particularly valuable for both clinical practice and research within the Norwegian context. Given the prevalence of CB‐PTSD observed in our sample and the current absence of systematic assessment in Norwegian postpartum care, the City BiTS‐Nor serves as a crucial tool for identifying women who may require support after traumatic childbirth experiences. Alongside the ongoing validation of a partner version, this validation study significantly enhances the detection and understanding of CB‐PTSD in Norway.

## AUTHOR CONTRIBUTIONS

Silje Marie Haga, Susan Garthus‐Niegel, and Malin Eberhard‐Gran contributed to the study conception, design, and data collection. Luisa Bergunde and Lara Seefeld performed the statistical analyses. Susan Ayers contributed to study conception. The first draft of the manuscript was written by Silje Marie Haga, Luisa Bergunde, Lara Seefeld, and Susan Garthus‐Niegel. All authors read and approved the final manuscript.

## FUNDING INFORMATION

No funding was obtained for the design or delivery of this study.

## ETHICS STATEMENT

The authors assert that all procedures contributing to this work comply with the ethics standards of the relevant national and institutional committees on human experimentation and with the Helsinki Declaration of 1975, as revised in 2008. Ethics approval was granted (date of issue: September 17, 2021) by the Norwegian Regional Committees for Medical and Health Research Ethics (project 275370), and the study was preregistered at https://osf.io/stbqj/.

## Data Availability

The datasets used and/or analyzed during the current study are available from the corresponding author on reasonable request.
